# Use and Outcomes of Dexamethasone in the Management of Malignant Small Bowel Obstruction

**DOI:** 10.1097/AS9.0000000000000431

**Published:** 2024-05-06

**Authors:** Frank F. Yang, Elina Serrano, Kyle S. Bilodeau, Michael Weykamp, Caitlin J. Silvestri, Ashleigh C. M. Bull, Brenda Lin, Sara L. Schaefer, Colette Galet, Luis J. Garcia, Baraka Gitonga, David T. Kolodziej, Samantha Esposito, Molly Parker-Brigham, Rohan Luhar, Avinash Mamgain, Kendrick C. Brown, Summer Dewdney, Thea P. Price, Nicole Siparsky, Sarah Knerr, Pauline K. Park, Sabrina Sanchez, Dionne A. Skeete, Katherine N. Fischkoff, David R. Flum

**Affiliations:** * From the Department of Surgery, University of Washington, Seattle, WA; † Department of Surgery, Columbia University Irving Medical Center, New York, NY; ‡ Department of Surgery, University of Iowa Carver College of Medicine, Iowa City, IA; § Department of Surgery, Boston University, Boston, MA; ‖ Department of Surgery, University of Michigan, Ann Arbor, MI; ¶ Department of Surgery, Rush University Medical Center, Chicago, IL; # Department of Health Systems and Population Health, University of Washington, Seattle, WA.

**Keywords:** dexamethasone, mSBO, nonoperative management

## Abstract

**Objective:**

To describe rates of dexamethasone use in the nonoperative management of malignant small bowel obstruction (mSBO) and their outcomes.

**Background:**

mSBO is common in patients with advanced abdominal-pelvic cancers. Management includes prioritizing quality of life and avoiding surgical intervention when possible. The use of dexamethasone to restore bowel function is recommended in the National Comprehensive Cancer Network guidelines for mSBO. Yet, it is unknown how often dexamethasone is used for mSBO and whether results from nonresearch settings support its use.

**Methods:**

This is a multicenter retrospective cohort study including unique admissions for mSBO from January 1, 2019 to December 31, 2021. Dexamethasone use and management outcomes were summarized with descriptive statistics and multiple logistic regression.

**Results:**

Among 571 admissions (68% female, mean age 63 years, 85% history of abdominal surgery) that were eligible and initially nonoperative, 26% [95% confidence interval (CI) = 23%–30%] received dexamethasone treatment (69% female, mean age 62 years, 87% history of abdominal surgery). Dexamethasone use by site ranged from 13% to 52%. Among dexamethasone recipients, 13% (95% CI = 9%–20%) subsequently required nonelective surgery during the same admission and 4 dexamethasone-related safety-events were reported. Amongst 421 eligible admissions where dexamethasone was not used, 17% (95% CI = 14%–21%) required nonelective surgery. Overall, the unadjusted odds ratio (OR) for nonelective surgery with dexamethasone use compared to without its use was 0.7 (95% CI = 0.4–1.3). Using multiple logistic regression, OR after adjusting for site, age, sex, history of abdominal surgery, nasogastric tube, and Gastrografin use was 0.6 (95% CI = 0.3–1.1).

**Conclusion:**

Dexamethasone was used in about 1 in 4 eligible mSBO admissions with high variability of use between tertiary academic centers. This multicenter retrospective cohort study suggested an association between dexamethasone use and lower rates of nonelective surgery, representing a potential opportunity for quality improvement.

## Abstract 2

Mini-abstract:

In this multicenter retrospective study, dexamethasone was used in 1 in 4 eligible admissions per National Comprehensive Cancer Network guidelines for malignant small bowel obstruction (mSBO). In adjusted analyses, there was a trend towards an association between dexamethasone use and fewer nonelective surgery. Dexamethasone may be an important adjunct to the nonoperative management of mSBO, representing an opportunity for quality improvement.

## INTRODUCTION

Malignant small bowel obstruction (mSBO) is a common and serious complication in advanced gastrointestinal or gynecologic cancer. Although the exact incidence is unknown, mSBO has been estimated to affect 3%–15% of all patients with cancer, with retrospective and autopsy studies suggesting prevalence rates of 5% to 51% of patients with ovarian malignancies and 10% to 28% of patients with gastrointestinal malignancies.^1–3^ The management of mSBO is distinct from benign small bowel obstruction in part because of patients’ decreased mobility and functional status, frequent lack of further chemotherapeutic options, and high mortality and morbidity associated with palliative surgery.^4^

The primary goal of mSBO management focuses on quality of life, including effective symptom control and avoiding surgery when possible.^5–7^ Management of mSBO on an individual level is often multidisciplinary with a combination of medical, surgical, or endoscopic options considered.^8^ Operative palliative intervention carries estimates of mortality of 9% to 40% and complication rates of 9% to 90% with overall survival from 3 to 6 months.^9,10^ Symptomatic improvement, gain of gastrointestinal function, or quality of life following surgical intervention for mSBO had rarely been assessed or reported in the literature, although there is growing interest in such outcomes.^4,11,12^ The SWOG S1316 trial is a recently published prospective randomized control trial comparing surgical and nonoperative management of mSBO with preliminary results suggesting no difference in key outcomes, including 90-day survival, hospital length-of-stay, and days requiring nasogastric tube (NGT) decompression, between groups randomized to surgery or nonoperative management.^13,14^ Therefore, there is a significant interest in optimizing adjunct nonoperative measures to manage mSBO, specifically corticosteroid use.^15^

In a Cochrane review meta-analysis last updated in 2016, including data from 3 small randomized trials, patients with mSBO who receive dexamethasone had an odds ratio (OR) of 0.51 [95% confidence interval (CI) = 0.21–1.23] for unresolved bowel obstruction compared to placebo. The authors concluded that there was a trend for evidence that dexamethasone given intravenously at a dose ranging from 6 to 16 mg may bring about the resolution of bowel obstruction. Additionally, the incidence of corticosteroid-related side effects in all included studies was extremely low.^4,16,17^

Although the National Cancer Consortium Network (NCCN) recommended dexamethasone for mSBO in its 2009 palliative care guidelines, there is no current data on the nationwide prevalence of dexamethasone use in mSBO management.^18^ Furthermore, the extent of dexamethasone use and outcomes for mSBO in nonresearch settings has not been studied. Herein, we undertook a multi-institutional retrospective cohort study assessing dexamethasone use for patients with mSBO and assessed outcomes.

## METHODS

### Ethical Statement

This study was approved by each center’s institutional review board.

### Study Design and Patient Selection

This is a multicenter retrospective cohort study. Unique admissions for mSBO at 6 academic centers (Boston Medical Center, Columbia University, Rush University, University of Iowa, University of Michigan, and University of Washington) from January 1, 2019 to December 31, 2021 were included. Cohort identification and data abstraction were uniform across sites. Diagnoses of or suspicion for mSBO were noted if the patient had radiographic or clinical evidence of small bowel obstruction secondary to new, known, or suspected malignant disease per clinician notes within the electronic health record. Patients were considered eligible for dexamethasone if they were admitted for mSBO, underwent initial nonoperative management, and had no contraindication for dexamethasone such as active infection, shock, hyperglycemia, or previous intolerance to dexamethasone. Patients were excluded if the suspected obstruction was proximal to the ligament of Treitz or within the large bowel. Dexamethasone use was noted if at least one dose of IV dexamethasone was given for any indication during nonoperative management while inpatient. Nonelective operative intervention was noted if the patient underwent surgery at any point following initial nonoperative management, exclusive of prophylactic or planned gastrostomy.

### Data Collection

Patient electronic medical records were reviewed. Demographics, comorbidities, cancer type, admission length-of-stay, nasogastric tube use, Gastrografin small bowel follow-through (SBFT) use, dexamethasone use, contraindications to dexamethasone use, operative interventions, endoscopic or interventional radiology (IR) interventions, and dexamethasone safety events were collected.

### Statistical Analysis

Primary outcomes included rates of dexamethasone use for mSBO and rates of nonelective operative intervention. Outcomes were summarized with descriptive statistics, including measures of central tendency and variability, means (standard deviation), frequencies (%), and 95% CIs were calculated using the Wilson score interval method. Adjustments for age, sex, history of abdominal surgery, site (hospital-specific covariate), nasogastric decompression, and Gastrografin SBFT use were made using multiple logistic regression analysis with reported OR and 95% CIs. Given this was a multicenter design, a hospital-specific covariate was included within multivariate models to account for hierarchical clustering of patients within hospitals and thus account for any variance within each respective hospital system. All statistical analysis was performed using RStudio Statistical Software (version 2022.07.2 + 576., R Core Team 2022).

## RESULTS

### Patient Selection

There were 498 patients with 644 unique admissions for mSBO from 2019 to 2021 across 6 academic centers. Seventy-three admissions were excluded because they were ineligible for dexamethasone, including 49 who underwent immediate surgical intervention and 24 who did not tolerate dexamethasone previously, were septic at presentation, or transitioned to comfort care immediately at presentation.

### Population Characteristics

A total of 571 admissions were initially managed nonoperatively and patients were eligible for dexamethasone use per NCCN guidelines, with sociodemographic factors shown in Table 1. The most common primary cancer type was those of gynecological origin (36%), followed by gastrointestinal tract distal to the ligament of Treitz (GI) (25%), foregut (9%), hepatopancreaticobiliary (9%), and genitourinary (7%).

**TABLE 1.** Baseline Characteristics of Patients Presenting With Malignant Small Bowel Obstruction Who Undergo Initial Nonoperative Management Following Admission

	Total Admissions (n = 571) No. (%)	No Dexamethasone (n = 421) No. (%)	Dexamethasone (n = 150) No. (%)
Age—years (mean ± standard deviation)	63 ± 13	63 ± 13	62 ± 12
Sex Female Male	391 (68) 180 (32)	287 (68) 134 (32)	104 (69) 46 (31)
Cancer type Foregut Hepatopancreaticobiliary Gastrointestinal Gynecologic Genitourinary Blood Other Unknown	49 (9) 54 (9) 145 (25) 204 (36) 40 (7) 28 (5) 41 (7) 10 (2)	22 (5) 43 (10) 120 (29) 143 (34) 29 (6) 22 (5) 29 (6) 8 (2)	22 (15) 11 (7) 25 (17) 61 (41) 11 (7) 6 (4) 12 (8) 2 (1)
History of prior abdominal surgery	484 (85)	353 (84)	131 (87)

### mSBO Management

**FIGURE 1.**
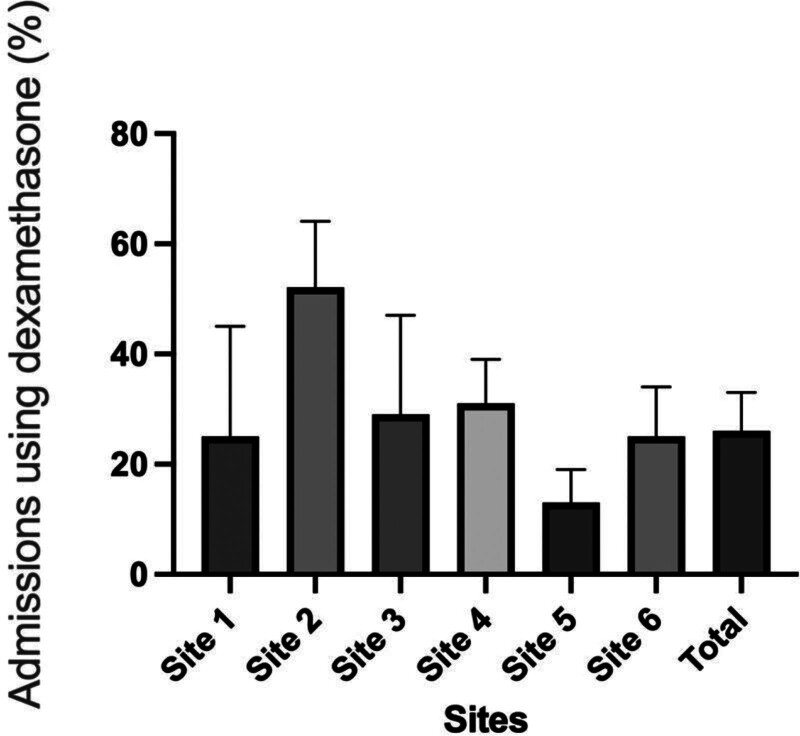
Variability among sites for dexamethasone use among eligible admissions.

Of eligible admissions, 150, or 26% (95% CI = 23%–30%), received dexamethasone treatment. The mean age of this population was 62 years old, 69% were female, and 87% had a history of prior abdominal surgery. A total of 421 patients did not receive dexamethasone, with a mean age of 63 years old, 68% were female, and 84% had a history of prior abdominal surgery. Between sites, the rate of dexamethasone use for eligible admissions varied between 13% and 52% (Fig. 1). There were no sociodemographic differences between those who received and those who did not receive dexamethasone.

Of the 150 admissions where dexamethasone was given, 112 also received nasogastric tube decompression (75%, 95% CI = 67%–81%) and 18 received Gastrografin SBFT (12%, 95% CI = 8%–18%). There were 4 dexamethasone-related safety-events reported, including 2 cases of hyperglycemia, one for concern for new infection, and one for hallucinations. Among admissions where dexamethasone was given, there were 20 (13%, 95% CI = 9%–20%) nonelective operative interventions (exclusive of planned gastrostomy), 5 (3%, 95% CI = 1%–8%) operative gastrostomies, and 34 (23%, 95% CI = 17%–30%) endoscopic or IR-placed gastrostomy or stenting procedures. The mean length-of-stay was 11.8 days (95% CI = 10.2–13.4), the proportion of patients requiring a readmission for mSBO following an admission where dexamethasone was used was 14% (95% CI = 9%–21%), and the mean number of readmissions following an admission where dexamethasone was given was 0.16 (95% CI = 0.9–0.24).

Of the 421 admissions where dexamethasone was not used, 270 received nasogastric tube decompression (64%, 95% CI = 59%–69%), 95 received Gastrografin SBFT (23%, 95% CI = 18%–27%). There were 72 (17%, 95% CI = 14%–21%) nonelective operative interventions, 6 (1%, 95% CI = 0%–3%) operative gastrostomies, and 73 (17%, 95% CI = 14%–21%) endoscopic or IR-placed gastrostomy or stenting procedures. The mean length-of-stay was 8.4 days (95% CI = 7.5–9.3), the proportion of patients requiring a readmission for mSBO following an admission where dexamethasone was used was 15% (95% CI = 11%–19%), and the mean number of readmissions amongst those who never received dexamethasone for the treatment of mSBO was 0.21 (95% CI = 15%–27%).

**FIGURE 2.**
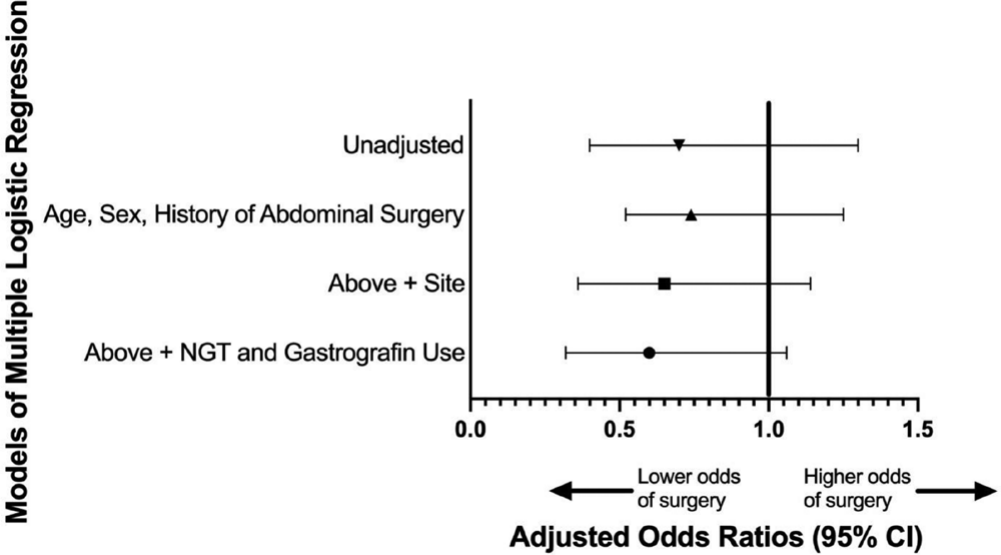
Unadjusted and adjusted odds ratio for nonelective operative intervention with dexamethasone use. Lower OR for nonelective operative intervention with dexamethasone use following covariate adjustments.

Overall, the absolute risk reduction of nonelective operative intervention with dexamethasone use was −4% (95% CI = −10%−3%) (Table 2). The unadjusted OR for nonelective operative intervention with dexamethasone use compared to without dexamethasone was 0.7 (95% CI = 0.4–1.3). Using multiple logistic regression, the OR after adjusting for site, age, sex, history of abdominal surgery, NGT, and Gastrografin SBFT use was 0.6 (95% CI = 0.3–1.1) (Fig. 2).

**TABLE 2.** Rates of Nonelective Operative Intervention Following Nonoperative Management of Malignant Small Bowel Obstruction Amongst Those Who Received Dexamethasone, Stratified by Site

	Rates of Nonelective Operative Intervention			
	No Dexamethasone—% (95% CI)	Dexamethasone—% (95% CI)	Unadjusted Odds Ratios for Nonelective Operative Intervention With Dexamethasone–OR (95% CI)	Adjusted Odds Ratios for Nonelective Operative Intervention With Dexamethasone—OR (95% CI)
Site 1	22% (9–45)	0% (0–50)	N/A*	N/A*
Site 2	27% (15–44)	11% (4–25)	0.3 (0.1–1.2)	0.3 (0.1–1.4)†
Site 3	36% (20–57)	33% (12–65)	0.9 (0.2–4.5)	0.5 (0.1–3.2)†
Site 4	14% (9–22)	13% (6–25)	0.9 (0.3–2.4)	0.7 (0.2–1.9)†
Site 5	13% (9–19)	12% (4–30)	0.9 (0.2–3.2)	1.0 (0.2–3.2)†
Site 6	18% (11–28)	15% (6–34)	0.8 (0.2–2.5)	1.0 (0.2–3.8)†
Total	17% (14–21)	13% (9–20)	0.7 (0.4–1.3)	0.6 (0.3–1.1)‡

* Model fails to converge given no operations were noted among those who underwent dexamethasone use.

† Adjusted for age, sex, history of abdominal surgery, nasogastric tube decompression, and Gastrografin small bowel follow-through use.

‡ Adjusted for site, age, sex, history of abdominal surgery, nasogastric tube decompression, and Gastrografin small bowel follow-through use.

Subgroup analysis did not demonstrate any effect size of dexamethasone use on the rate of nonelective operative intervention to be more pronounced amongst those with a history of prior abdominal surgery, those who received NGT decompression, Gastrografin SBFT, or gastrostomy placement, or for any of the 5 most common cancer types noted (Supplemental Table 1, see http://links.lww.com/AOSO/A344),

## DISCUSSION

In this multi-institutional retrospective cohort study, dexamethasone was used in only 1 in 4 eligible admissions for mSBO with high variability of its use between institutions. After adjusting for site, age, sex, history of prior abdominal surgery, and other adjuncts of nonoperative management such as NGT and Gastrografin SBFT, the use of dexamethasone was associated with fewer nonelective operative interventions.

One of the major challenges of this study revolves around the complexity of multiple clinical endpoints that are possible for patients with mSBO. In this study, we selected nonelective operations as the primary outcome given their clinical relevance and importance and their association with high morbidity and mortality. Resolution of bowel obstruction has been used as an endpoint in other studies. However, this is difficult to definitively determine retrospectively given that many patients undergo no procedure and are able to become discharged from the hospital but are discharged without full tolerance to a normal diet, have nutrition supplemented with enteral tube feeds or partial or full TPN, or enter into hospice care and die shortly after discharge.

Another challenge is accounting for the therapeutic intent for gastrostomies, either operative or endoscopic, whether they be placed to avoid a larger operation, or prophylactically in a more elective setting if there is high clinical suspicion for recurrence. The OR after adjusting for site, age, sex, history of abdominal surgery, NGT, Gastrografin, and combined surgical gastrostomy and endoscopic enteral access or stenting procedures were similar [0.6 (95% CI = 0.3–1.1)]. However, gastrostomies and endoscopic procedures were not included in the final logistic regression model given that the timing and intent of many of these procedures, whether prophylactic or planned in cases of high clinical suspicion for future recurrence of mSBO, were difficult to determine in this dataset. There was variation between institutions for rates of gastrostomy placements, although, with subgroup analysis and regression modeling, it was not a driver of our effect size.

The use of corticosteroids in palliative therapy is not novel and has been prescribed as part of palliative management for patients with advanced malignancy since the 1950s for its central antiemetic, anti-inflammatory, antisecretory, and analgesic effects.^15^ mSBO can be caused by bowel-occlusive intramural infiltration, extrinsic compression of the bowel, functional motility disorders, or side effects of radiotherapy due to stricture and negative impact on peristalsis.^19^ Common pathophysiology to each process is the accumulation of fluid and gases via a cascade of inflammatory mediator release.^15,20^ It is thought that steroids target the inflammatory mediator cascade and may be a potential treatment for mSBO by decreasing gut wall edema, peritoneal inflammation, and inflammation in proximity to the obstruction while indirectly decreasing pain via relief of luminal obstruction.^15,21^

A 1999 meta-analysis and a 2000 Cochrane review found a near doubling of the rate of nonoperative resolution of mSBO with the use of corticosteroids, concluding there was “evidence that dexamethasone… may bring about the resolution of bowel obstruction.”^16,17^ These reviews included 3 randomized, placebo, double-blind controlled trials (a total of 89 patients)^22,^^23^ and 7 retrospective and prospective reports evaluating dexamethasone 5–15 mg/day.^17^ Meta-analysis of the randomized trials found an OR of 0.51 (with a confidence interval spanning 1) for unresolved mSBO after administration of dexamethasone.^4^ In the largest of the 3 studies included, Laval et al^24^ evaluated 58 French patients with advanced abdominopelvic malignancy and small bowel obstruction. Of the 40 patients without a nasogastric tube, symptoms were relieved in 68% of cases versus 33% among placebo-treated patients (*P* = 0.047).^24^ In the 12 patients with a nasogastric tube, symptoms were relieved in 60% versus 33% (*P* = 0.080).^24^ Most recently, a 2021 UK single-center retrospective study of the use of corticosteroids in mSBO showed that patients in whom dexamethasone was given were more likely to resolve without surgery [24% of patients who received dexamethasone proceeded to surgery compared to 60% who were not given dexamethasone and later proceeded to surgery (*P* < 0.001)].^25^ However, since the inclusion of dexamethasone within NCCN’s palliative guidelines for the management of mSBO in 2009, the rate of its use in nonresearch settings has not been evaluated. This study aimed to evaluate its use and effectiveness across multiple tertiary centers. Using the effect size suggested by this data, a randomized control trial comparing dexamethasone with placebo would require over 2800 patients. Given concerns over the feasibility and cost of such an RCT, future evaluation of dexamethasone may be limited to retrospective data.

Of note, 85% of patients included in this study have a history of prior abdominal-pelvic surgery. The specific etiology of SBO in patients with a known or new diagnosis of abdominal malignancy may be presumed to be malignant. However, in patients with prior surgery, adhesive SBO may be difficult to differentiate from mSBO with radiographic evidence alone and the ultimate diagnosis sometimes may only be confirmed in the operating room. With some elements of shared pathophysiology of extrinsic compression of the bowel, negative impacts to peristalsis, and accumulation of fluid and gases associated with a cascade of inflammatory mediator release, adhesive SBO may represent an opportunity for evaluation of further uses of corticosteroids as an adjunct to nonoperative bowel obstruction management.

One limitation is confounding by indication for dexamethasone use and the inability to otherwise adjust for disease severity in admissions that are initially nonoperative. Although we attempted to address confounding by indication by adjusting for certain confounders, our retrospective study design precluded us from accounting for all or unknown confounders. Similarly, we attempted to adjust for clustering of data through the inclusion of a site-specific covariate, but our model choice, although appropriate for our research question, does not allow for out-of-sample predictions compared to a mixed-effects model and therefore may not be generalizable to all patients with mSBO. Finally, our sample size is such that our effect size showed a trend, but was not large enough to demonstrate statistical significance.

## CONCLUSION

In summary, dexamethasone was used in 1 in 4 eligible admissions for the management of mSBO per NCCN guidelines, with high variability between centers. After adjusting for sociodemographic and clinical factors, the use of dexamethasone was associated with fewer nonelective operative interventions [OR 0.6 (95% CI = 0.3–1.1)]. Overall, dexamethasone may be an important adjunct to the usual nonoperative management of mSBO and represents a potential opportunity for quality improvement.

## Supplementary Material


