# Supplementary material for: Use and Outcomes of Dexamethasone in the Management of Malignant Small Bowel Obstruction
Source: Ann Surg Open. 2024 May 6;5(2):e431. doi: 10.1097/AS9.0000000000000431 (PMC11191995; doi:10.1097/AS9.0000000000000431)
Supplement: Supplementary file 1 [file as9-5-e431-s001.pdf]

Supplementary Table 1. Subgroup analysis of adjusted odds ratios for non-elective operative intervention with dexamethasone use.

	No dexamethasone (n=421)	Dexamethasone (n=150)	Adjusted OR for non-elective operative intervention with dexamethasone
History of prior surgery			
History of abdominal surgery	354 (84%)	132 (88%)	0.6 (0.3-1.2)[1]
No history of abdominal surgery	67 (16%)	18 (12%)	0.3 (0.0-1.4)[1]
Management factors			
Gastrografin SBFT – no. (%)	95 (23%)	18 (12%)	0.7 (0.1-2.7)[2]
NGT – no. (%)	270 (64%)	112 (75%)	0.6 (0.3-1.1)[3]
Combined prophylactic gastrostomy or endoscopic PEG/stent – no. (%)	79 (19%)	38 (25%)	1.1 (0.2-5.3)[4]
Cancer type			
Foregut – no. (%)	22 (5%)	22 (15%)	2.6 (0.0-442)[4]
HPB – no. (%)	43 (10%)	11 (7%)	3.8 (0.0-16)[4]
GI – no. (%)	120 (29%)	25 (17%)	0.3 (0.0-1.4)[4]
Gyn – no. (%)	143 (34%)	61 (41%)	1.0 (0.4-2.6)[4]
GU – no. (%)	29 (6%)	11 (7%)	0.1 (0.0-3.1)[4]

[1]Adjusted for age, sex, site, NGT, and Gastrografin SBFT use

[2]Adjusted for age, sex, history of abdominal surgery, site, and NGT use

[3]Adjusted for age, sex, history of abdominal surgery, site, and Gastrografin SBFT use

[4]Adjusted for age, sex, history of abdominal surgery, site, NGT, and Gastrografin SBFT use
